# Will Coral Islands Maintain Their Growth over the Next Century? A Deterministic Model of Sediment Availability at Lady Elliot Island, Great Barrier Reef

**DOI:** 10.1371/journal.pone.0094067

**Published:** 2014-04-23

**Authors:** Sarah Hamylton

**Affiliations:** School of Earth and Environmental Sciences, University of Wollongong, Wollongong, New South Wales, Australia; University of Vigo, Spain

## Abstract

A geomorphic assessment of reef system calcification is conducted for past (3200 Ka to present), present and future (2010–2100) time periods. Reef platform sediment production is estimated at 569 m^3^ yr^−1^ using rate laws that express gross community carbonate production as a function of seawater aragonite saturation, community composition and rugosity and incorporating estimates of carbonate removal from the reef system. Key carbonate producers including hard coral, crustose coralline algae and *Halimeda* are mapped accurately (mean R^2^ = 0.81). Community net production estimates correspond closely to independent census-based estimates made *in-situ* (R^2^ = 0.86). Reef-scale outputs are compared with historic rates of production generated from (i) radiocarbon evidence of island deposition initiation around 3200 years ago, and (ii) island volume calculated from a high resolution island digital elevation model. Contemporary carbonate production rates appear to be remarkably similar to historical values of 573 m^3^ yr^−1^. Anticipated future seawater chemistry parameters associated with an RCP8.5 emissions scenario are employed to model rates of net community calcification for the period 2000–2100 on the basis of an inorganic aragonite precipitation law, under the assumption of constant benthic community character. Simulations indicate that carbonate production will decrease linearly to a level of 118 m^3^ yr^−1^ by 2100 and that by 2150 aragonite saturation levels may no longer support the positive budgetary status necessary to sustain island accretion. Novel aspects of this assessment include the development of rate law parameters to realistically represent the variable composition of coral reef benthic carbonate producers, incorporation of three dimensional rugosity of the entire reef platform and the coupling of model outputs with both historical radiocarbon dating evidence and forward hydrochemical projections to conduct an assessment of island evolution through time. By combining several lines of evidence in a deterministic manner, an assessment of changes in carbonate production is carried out that has tangible geomorphic implications for sediment availability and associated island evolution.

## Introduction

Reef islands are low-lying accumulations of biogenically derived sediments [1]. They are important for a range of socio-economic and ecological reasons, including the provision of habitable land in low-lying countries (e.g. the Maldives, Torres Strait, Tuvalu, Kiribati and the Marshall Islands) [2], nesting ground for turtles [3] and habitat for terrestrial amphibians, mammals and reptiles [4]. They also generate income from island-related tourism, for example, a revenue of five billion was generated for the Australian economy from tourism activities largely related to the islands of the Great Barrier Reef Marine Park in 2012 [5].

Reef islands are constructed from unconsolidated sediments derived from benthic calcium carbonate producers such as scleractinian corals, coralline algae, green calcified algae, molluscs and benthic foraminifera [6]. These communities are sometimes termed the “biological sediment factory” [7]; they colonise the reef platform upon which islands form and are broken down and reworked by waves and currents and deposited over time to form the sedimentary island landform. Under optimal conditions, a reef island will continue to increase in size until it reaches an equilibrium, whereby sediment delivery and removal are balanced [8], [9].

Because reef island sediments derive entirely from surrounding coral reef and reef flat environments, they are highly sensitive to environmental conditions that may modify reef community composition and productivity, including sea surface temperature [10], [11], seawater chemistry, particularly aragonite saturation state [12]–[18], ambient light levels [19], [20], salinity [21], reef growth-sea level interactions [22] and anthropogenic influences, such as nutrient inputs [13], [23]–[25]. In the Caribbean, regional shifts in species composition of shallow forereef habitat, including reductions in live coral cover, have led to carbonate production rates that are at least 50% lower than historical Holocene values, with accompanying transitions to net erosional carbonate budgets [26]. The prognosis for future calcification rates is bleak, with chemical projections of localised sample sites suggesting that reef communities may effectively start to dissolve over the coming century [27], [28]. How this sensitivity will translate into island dynamics, through fluctuations in sediment supply and associated accretion rates is less evident. Different coral reef settings (location, geomorphic zones) vary greatly in the rates at which they produce calcium carbonate and how such environmental modifications will impact reef island sediment supply and geomorphic stability remains a critical but poorly resolved question. This is particularly the case at spatial scales that have geomorphic meaning, i.e. the carbonate production of complete reef systems and the island deposition that they support.

The objective of the present study is to conduct a geomorphic assessment of reef system calcification and associated island evolution for past (3200 Ka to present), present and future (2010–2100) time periods at Lady Elliot Island (southern Great Barrier Reef). This is achieved through a combination of geospatial analysis, hydrochemical measurements and radiocarbon evidence. Contemporary levels of carbonate production are estimated for the complete reef system by mapping the magnitude and distribution of key benthic community calcifiers across the reef platform. This information is then utilised alongside estimates of reef surface rugosity in a rate law calculation to estimate net community carbonate production from inorganic aragonite precipitation. Results are validated against independent *in-situ* census based estimates of carbonate production. Contemporary estimates of carbonate production are then compared to historical levels of island accretion, ascertained through a combination of *i*. radiocarbon evidence to identify the point at which island deposition began, and *ii*. the development of a high resolution digital elevation model of the island, such that an approximation of of average island accretion rate can be derived by dividing island volume by age. Finally, hydrochemical measurements taken from the reef flat are used to simulate future levels of carbonate production for the complete reef system using anticipated values of aragonite saturation under a RCP8.5 emissions scenario. Simulations are undertaken for 4 seasonal periods (summer, autumn, winter, spring) and calculated across a complete tidal cycle to account for natural chemical fluctuations. Such an assessment is of particular interest because Lady Elliot Island is the southernmost coral Cay of the Great Barrier Reef and is therefore representative of an island site at the margins of environmental conditions (e.g. sea surface temperatures) that are suitable for carbonate production.

## Methods

### Site description

**Figure 1:**
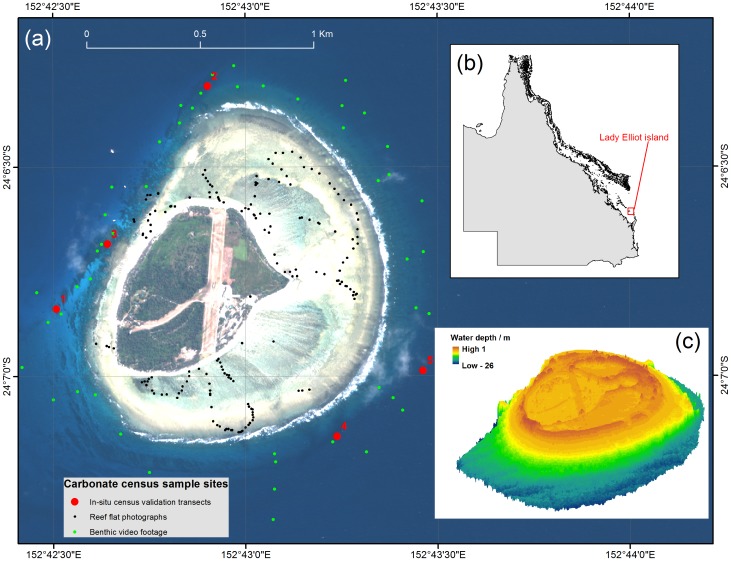
Fieldwork undertaken at Lady Elliot Island, Great Barrier Reef (24°07′S :152°43′E). (a) A Quickbird satelite image of Lady Elliot Island, showing sample sites for the carbonate production census (red dots, numbering relates to sites detailed in Table 2), reef flat and reef slope ground referencing points (black and green dots) (b) The location of Lady Elliot Island along the Queensland coastline, at the southern end of the Great Barrier Reef, (c) a digital elevation model of Lady Elliot island and the surrounding reef platform.

At 24°07′S and 152°43′E, Lady Elliot island lies 84 km offshore from the Queensland coastline and is part of the Capricorn-Bunker group (Figure 1). Lady Elliot Island is a shingle deposit (area 0.43sq km) comprised of coral rubble and carbonate sand bound by guano derived phosphatic cement and stabilised by beachrock along its eastern perimeter. Radiocarbon dating of prograded shingle ridge sequences found that island deposits have accumulated laterally at nearly uniform rates since about 3200 years B.P., with maximum mean lateral accumulation rates of 90 m Ka^−1^ and 60 m Ka^−1^ for the windward and leeward sequences respectively [29]. The island has formed on the central to western side of a reef platform (platform area 1.43sq km). Along the eastern coastline this platform has a well-developed continuous forereef slope that is approximately 210 m wide from reef crest to a lower forereef (at a depth of around 15 m), with distinct spur and groove morphology. Fringing reef is patchier along the western, leeward side of the island with less distinct geomorphic zonation. Here, the reef forms a series of coral patches that extend along a south-western trajectory for approximately 500 m at a depth of 8–15 m from the centre of the reef platform.

Lady Elliot Island is subject to a diurnal mesotidal regime with a mean spring tidal range of 1.7 m and a neap tidal range of 0.9 m [30]. Offshore waters surrounding the reef platform are 30–40 m deep [31]. Prevailing winds are from the southeast in the early part of the year (January to May), with southerlies dominating in winter (June to September). The wave climate of the southern GBR is characterised by deepwater swell [2] with storm and wind driven waves [32].

### An inventory of the Lady Elliot Island carbonate budget

**Figure 2:**
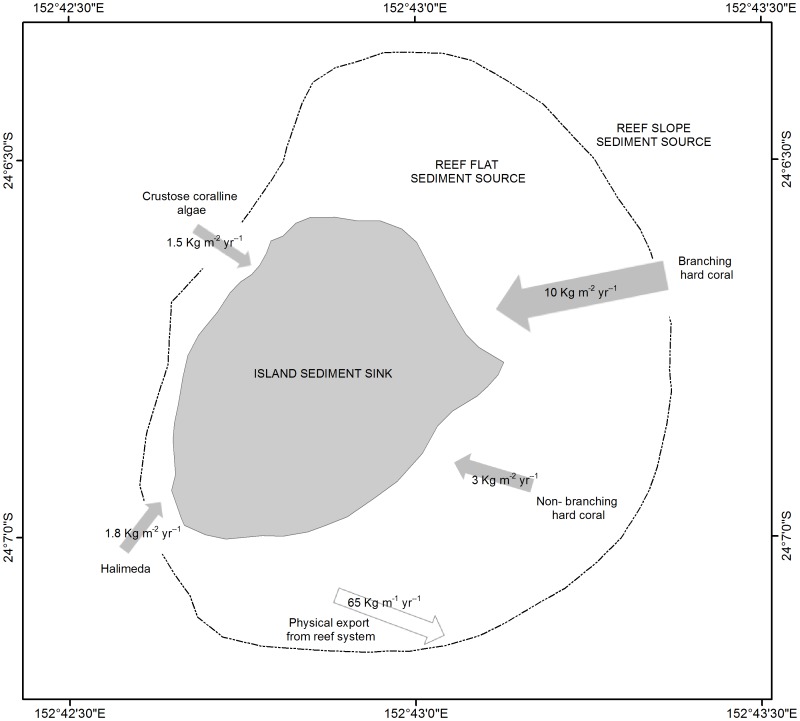
Conceptual model of the carbonate budget for Lady Elliot Island.

A detailed inventory of the carbonate budget components for Lady Elliot island was undertaken to develop an initial conceptual model in order to constrain the relationship between reef benthic carbonate production and sediment delivery to the island, and ascertain the applicability of the *in-situ* census based methods for estimation of reef carbonate production within a Pacific context (Figure 2). The inventory identified four key carbonate producers included branching hard coral, hard coral (non-branching), crustose coralline algae and *Halimeda*. An extensive survey of the reef flat, including foraminifera and mollusk population surveys at 218 locations, indicated negligible contribution from these communities to the carbonate budget. In addition, no dense macroalgal beds were observed on the reef flat or around the island platform (<25 m water depth). The only calcified macroalgae observed was *Halimeda*, which was observed in clusters of small patches (<1 m^2^), rather than dense beds.

Framework erosion by internal borers including endolithic organisms such as bivalves and worms and substrate grazers such as urchins was either entirely absent or observed in such low quantities that their influence was inconsequential. Parrotfish had a more localized influence, being absent from most surveys but with schools of up to 17 bullethead parrotfish observed at in the northwest (site 2, Figure 1). Bioerosive processes serve to break down carbonate framework and reduce it to sediment that either remains in-situ, is transported elsewhere in the island system, or exported off the reef slope. Given that the objective of the present study is to estimate the volume of sediment available for island maintenance and growth, it was therefore important to quantify how much carbonate was produced and how much was exported from the system altogether. For this purpose, sediment traps were deployed to estimate the rate at which sand and gravel sediments were exported off the reef slope (see section on estimating community carbonate production). This conceptual model did not attempt to define the processes responsible for the conversion of carbonate into sediments, rather it assumed that all carbonate sediments reaching the island were derived from the reef platform [7] and that a first order approximation of their annual volume could be calculated as the difference between the total carbonate produced across the entire reef platform and that exported from the system [33].

### Mapping the carbonate producers around Lady Elliot island (hard coral branching and non-branching, crustose coralline algae and Halimeda)

This work was undertaken with permission from the Great Barrier Reef Marine Park Authority (permit ref. G36002.1). Fifty snapshots of oblique underwater video footage of benthic cover were collected from a boat around Lady Elliot Island (Figure 1). Fugawi(TM) navigational software was used to locate the boat in real-time across a QuickBird satellite image of the island and surrounding reef platform. Points of interest were identified where benthic features or changes in image reflectance were apparent and an underwater video camera was lowered on a cable from the boat. The camera was held to record for 30 seconds drifting approximately 20 cm above the sea floor at an oblique angle. This generated an image with an instantaneous field of view of approximately 2 m^2^ that incorporated a side profile of the reef community, including the understorey. The video camera was mounted on a 50 m length of cable, permitting the deeper reef platform to be sampled after attaching weights to the base of the camera head. The geographical position of each video sample point was recorded with a dGPS (accuracy <1 m).

The distribution of four major carbonate producing benthic components (branching hard coral, hard coral (non-branching), crustose coralline algae and *Halimeda*) was mapped using a predictive habitat mapping approach [34], [35]. For the 50 video sample points, a statistical relationship was established between the cover of each benthic component and a series of independent physical variables across the reef platform [36]. Independent variables included remotely sensed benthic reflectance extracted from the QuickBird satellite image of the reef platform (bands 1 and 2), water depth derived from a digital elevation model (DEM) of the reef platform and the benthic terrain variables of slope and terrain rugosity. Reef rugosity was calculated from a digital elevation model of the reef platform following the derivation from Nellemann & Cameron [37]. The digital elevation model (Figure 1) was generated from a QuickBird multispectral satellite image of the reef using band ratio techniques [38] and validated against a single beam echo sounder dataset of 73,000 depth readings collected *in-situ*. The bathymetric echo sounder survey was undertaken with a Ceeducer Pro system (Frequency 30 KHz, ping rate 6 Hz) with an integrated GPS. Soundings were undertaken following a zig-zag pattern across the reef platform profile around the entire island. The tidal signal was removed from the depth readings using Navy Seafarer tides to convert all readings to a datum of mean sea level.

For each digital pixel of a raster grid covering the entire reef platform, a spatially explicit regression model predicted the distribution of each carbonate producer on the basis of the independent physical variables around Lady Elliot Island. This followed a methodology that has successfully modelled the distribution of major carbonate producers around Lizard Island on the northern GBR [39]. To establish the form of the regression equation from the 50 video sample points, values for the independent physical variables were extracted and regressed against the percentage of each carbonate-producing benthic component recorded at each site. The percentage cover of each benthic carbonate producer was then predicted across the entire reef platform around Lady Elliot by combining the beta coefficients with raster datasets representing the independent variables across the reef platform using the Model Builder and Raster Calculator tools of ArcGIS10.

### Estimating overall community carbonate production *G_net_* for the complete reef platform

Strong correlations between G_net_ values measured on reefs and rates of inorganic aragonite precipitation G_i_ were used as the basis for deriving a rate law with which net community calcification could be estimated on the basis of aragonite saturation state [27].
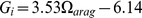
(1)


The inorganic aragonite precipitation rate law (Equation 1) was parameterised using recent hydrochemical measurements of aragonite saturation and temperature taken from the reef flat and slope at Lady Elliot Island across the four seasons [27], [40]. These accounted for dissolution where sediment-water exchange influenced the chemistry of the overlying seawater, although the relative importance of calcification or dissolution could not be partitioned. This relationship was strengthened by removing the daily signal of community primary production associated with the light/dark cycle and incorporating additional information on reef community character and rugosity. Following these adjustments, net community calcification was estimated across the complete reef platform from inorganic aragonite precipitation using the following rate law:
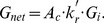
(2)


Where *A_c_* is a measure of the fraction of reef area actively depositing CaCO_3_ and *k^′^_r_* is the ratio between the total area of the reef actively precipitating CaCO_3_ and its planar area (i.e. the reef rugosity). *G_net_* was estimated in a deterministic manner by applying a modified version of the rate law on a per pixel basis. Different rates of inorganic aragonite precipitation were applied across the two reef zones by generating a reef flat and a reef slope mask (whereby pixels were either attributed a value of 1 if they belonged to the reef flat or 0 if not) and employing a conditional statement that inserted the correct G_i_ value depending on pixel location. In this way, the different aragonite conditions of the reef flat and slope could be accounted for. The term *A_c_* was adjusted to reflect the range of contributions made to *G_net_* by different community components. For each pixel, *A_c_* was estimated according to the relative contribution of the different carbonate producing community components and expressed as a proportion of the theoretical maximum calcification value. This was a pixel comprised completely of branching coral, the community component with the highest calcification rate (i.e. 10 Kg CacO_3_ m^−2^ yr^−1^):
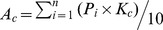
(3)where *P_i_* is the proportional cover of each community component and *K_c_* is the calcification rate of that community component for *n* community components. To convert values for overall carbonate production of the reef platform to island accretion, the volume of carbonate produced each year was reduced to account for sediment loss from the system.

To quantify the annual volume of sediment loss from the island, cylindrical sediment traps (35 cm length by 5 cm diameter) were deployed at the edge of the reef terrace (approximately 14 m water depth) adjacent to each of the in-situ census validation sites. In line with the model objective (to estimate sediment availability for island maintenance and growth), sediment loss was defined as any outward sediment movement beyond the boundary of the upper reef terrace and down the reef slope to a deeper position (>14 m), from which it is unlikely to be transported back up the reef flat and deposited on the island. A single sediment trap was deployed at each of the five sites (marked 1 to 5 on Figure 1) from June until November 2013 in line with protocols for the use of sediment traps in coral reef environments [41]. Upon recovery of the traps, the sand and gravel carbonate material in the traps was dried and weighed. The reef perimeter length was estimated to be 4395 m by digitising around the edge of the reef platform, along the reef crest, at a scale of 1∶500 over the geocorrected QuickBird image of the reef platform. Total average sediment loss around the reef perimeter was therefore estimated to be 65 kg m^−1^ yr^−1^, which was comparable with rates of off-reef sediment and rubble transport elsewhere [42]–[45].

### Validation of rate law with census-based estimates of carbonate production

To validate the modelled values of community carbonate production, calcification for the Lady Elliot island reef slope was measured *in-situ* using the *ReefBudget* census-based methodology [46], [47]. Surveys were conducted along transects at five sites around different aspects of the reef platform. This employed data on organism cover and abundance, alongside annual extension or production rates to determine net carbonate production rates (kg CaCO3 m^−2^ yr^−1^). This methodology yields continuous records of all cover types and transitions between different covers along the transect, thereby internally adjusting the sampling frequency on the basis of the spatial variation of the benthic cover itself. A detailed description of the methods, along with supporting datasets, field survey and data entry sheets can be downloaded from the ReefBudget website (http:/geography.exeter.ac.uk/reefbudget).

Two 10 m long transects were deployed running parallel to the reef crest, approximately 30 m apart at a depth of 10 m at each survey site. Coverage and dimensions of a range of carbonate producers within the reef community were recorded, including crustose coralline algae, hard corals of varying growth forms (branching, encrusting, massive, platy/foliose) and calcified macroalgae *Halimeda*. Substrate topographic complexity was incorporated to derive an accurate measure of the true surface area covered by each taxon by running a measuring tape over the substrate to record the length of transect conforming to the topography (d1) and simultaneously measuring the planar distance of transect covered. Rugosity (topographic complexity) of the survey site was then determined as d1/d2 [48], [23]. Several adaptations were made to the survey methodology to account for the differing nature of calcification dynamics in the Pacific. These included incorporation into the census the carbonate production rates for the calcified macroalgae *Halimeda* (1.80 Kg CacO_3_ m^−2^ yr^−1^, 48) and the substitution of calcification rates derived from the Pacific for branching and non-branching live coral (10 Kg CacO_3_ m^−2^ yr^−1^ and 3 Kg CacO_3_ m^−2^ yr^−1^ respectively, [49]) and crustose coralline algae (1.49 Kg CacO_3_ m^−2^ yr^−1^, [50]). Estimates of community calcification derived from *in-situ* surveys were compared against those derived using the rate law via regression for the ten transects surveyed by way of validation.

### Simulation of future *G_net_* for the complete reef platform (2010–2100)

To simulate values of *G_net_* for the complete reef platform, the *G_i_* term was adjusted to incorporate anticipated values of aragonite saturation for the summer, autumn, winter and spring periods at 10 year intervals from 2010 to 2100. Equation 2 was then implemented on a per-pixel basis, using the *G_i_* term calculated for anticipated future values of aragonite saturation state (Equation 1). Published values were adopted from the study conducted by Shaw et al., [27], in which aragonite saturation states were adjusted for the reef flat communities in line with pH fluctuations expected under the RCP8.5 emissions scenario, a high emissions scenario among the representative concentration pathways (RCP), with end-century atmospheric CO2 concentration of ∼900 ppm [51]. Aragonite saturation state values declined to a mean value of 2.13 (with an upper diurnal range limit of 3.95 and a lower limit of 0.85 by the end of the century, see Figure 1 of [52] for further detail).

The term A_c_ was not adjusted across simulation time intervals, which meant that the model assumed no change in the benthic community. Although changes in benthic community structure may arise due to lowered oceanic pH values, these would be difficult to predict for the community due to differential vulnerability within and between different taxonomical groups [53]. Furthermore, at Lady Elliot Island Shaw et al. [27] have observed metabolic processes (photosynthesis, respiration, calcification and dissolution) to give rise to extreme carbonate chemistry conditions in ponded reef flat water at low tide, which exceed aragonite conditions likely to occur by end-century in the open ocean [54]. Predicting the community response to ocean acidification therefore becomes further complicated by the need to incorporate natural diurnal variability in carbonate chemistry, as well as longer-term anthropogenic ocean acidification.

### Estimation of island volume

A digital elevation model of the island was constructed via an *in-situ* dGPS survey with a TrimbleRTK base station and rover unit. XYZ coordinates were collected for distinctive island features, including the low tide water line, the vegetation edge and along the strike and dip contours of the shingle ridges, as delineated in previous maps [29]. After post processing to achieve a higher spatial referencing accuracy (<1 m), the 2.3 million *xyz* point elevations were interpolated into a continuous surface using the natural neighbour algorithm within the spatial analyst interpolation toolbox of ArcGIS10. Total island volume was then calculated as the sum of the volume of the raster pixels in the grid. This approach assumed that the base of the island coincided with the lowest part of the DEM at the low tide line.

To estimate an approximate historical rate of island accretion, the total volume of the island was divided by the time elapsed since initial deposition began according to the available radiocarbon dating evidence [29]. Several assumptions were made for the purpose of this calculation, including that all carbonate material was derived from the reef platform, that the island has evolved under a regime of steady-state accumulation (this is likely not the case, although the average accretion rate may still be a valid reflection of total carbonate production) and that the earliest radiocarbon date of island sediments corresponds to the time of initial deposition. This latter assumption requires an explanation for the time lag between the reef platform reaching modern sea level around 6500 years ago and the onset of island deposition some 3200 years ago. This is provided by the fact that island growth will not commence until the reef platform is sufficiently established by coral growth and sedimentation to provide a robust foundation close to sea level [55].

## Results

Table 1 outlines average % cover observed for a range of reef community types from both the underwater video footage and the reef flat photographs. Overall, the dominant cover types were sand and crustose coralline algae, with live coral and dead coral observed in abundance on the reef flat.

**Table 1:** Average estimates of reef community cover for a range of different assemblages from the underwater video dataset (*n* = 50) and the reef flat photographs (*n* = 218).

Cover type	Underwater video (mean % cover estimations)	Reef flat photograph (mean % cover estimations)
Crustose coralline algae	30.89	15.29
Hard Coral (branching)	27.62	35.67
Hard Coral (encrusting)	8.41	22.82
Hard Coral (massive)	11.82	13.53
Hard Coral (platy/foliose)	10.50	8.03
Halimeda	21.25	8.25
Dead Coral	22.61	46.32
Rubble	15.34	27.43
Macroalgae	13.08	23.54
Calcareous macroalgae	10.00	31.56
Sand	30.59	52.05
Soft Coral	8.89	2.54
Turf algae	10.71	4.72

**Figure 3:**
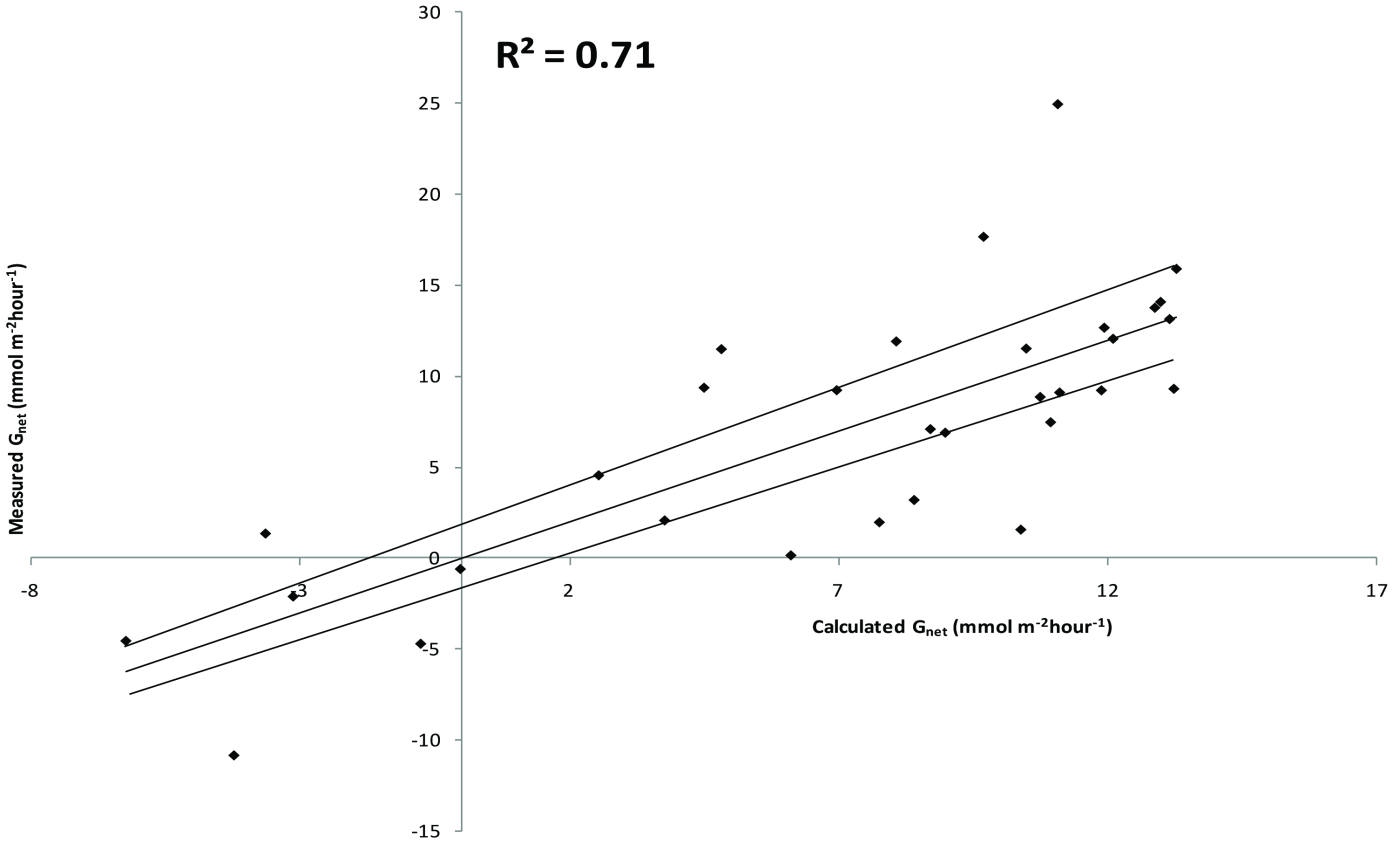
Calculated calcification (equation 2) vs. the corresponding measured net calcification for the reef flat hydrochemical samples at Lady Elliot Island. Dashed lines indicate error bars calculated from the standard error of the 30 samples with the analytical error associated with measurement of aragonite saturation state [25].

Figure 3 shows the calculated calcification (equation 2) vs. the corresponding measured net calcification for the reef flat hydrochemical samples at Lady Elliot Island. Dashed lines indicate error bars calculated from the standard error of the 30 samples with the analytical error associated with measurement of aragonite saturation state [25].

**Figure 4:**
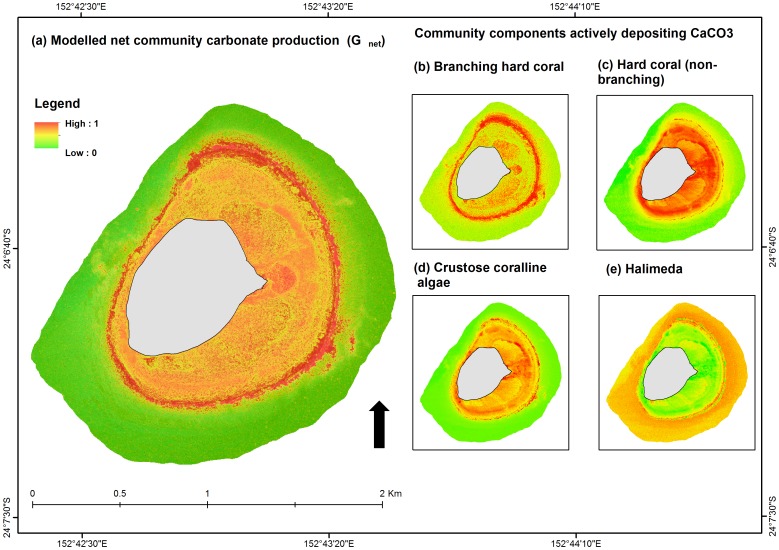
Digital maps of carbonate production at Lady Elliot Island, for the total community and the benthic cover of key benthic carbonate producers. (a) Net community carbonate production, G_net_ (Eqn. 2), (a) branching hard coral, (b) hard coral (non-branching), (c) crustose corralling algae, and (d) *Halimeda*. Note: Digital versions of these maps can be downloaded in raster format from the author's data archive at www.sarahhamylton/maps.

The map of reef area actively depositing CaCO_3_ at Lady Elliot Island suggested that the forereef slope was the most productive site (Figure 4a). Marked variation was evident in the distribution of the different carbonate producers, with branching hard corals dominating the upper reef slope, non-branching corals prevalent on the reef flat, crustose coralline algae inhabiting the reef flat and reef crest areas and *Halimeda* occupying deeper areas around the lower reef platform (Figure 4).

**Figure 5:**
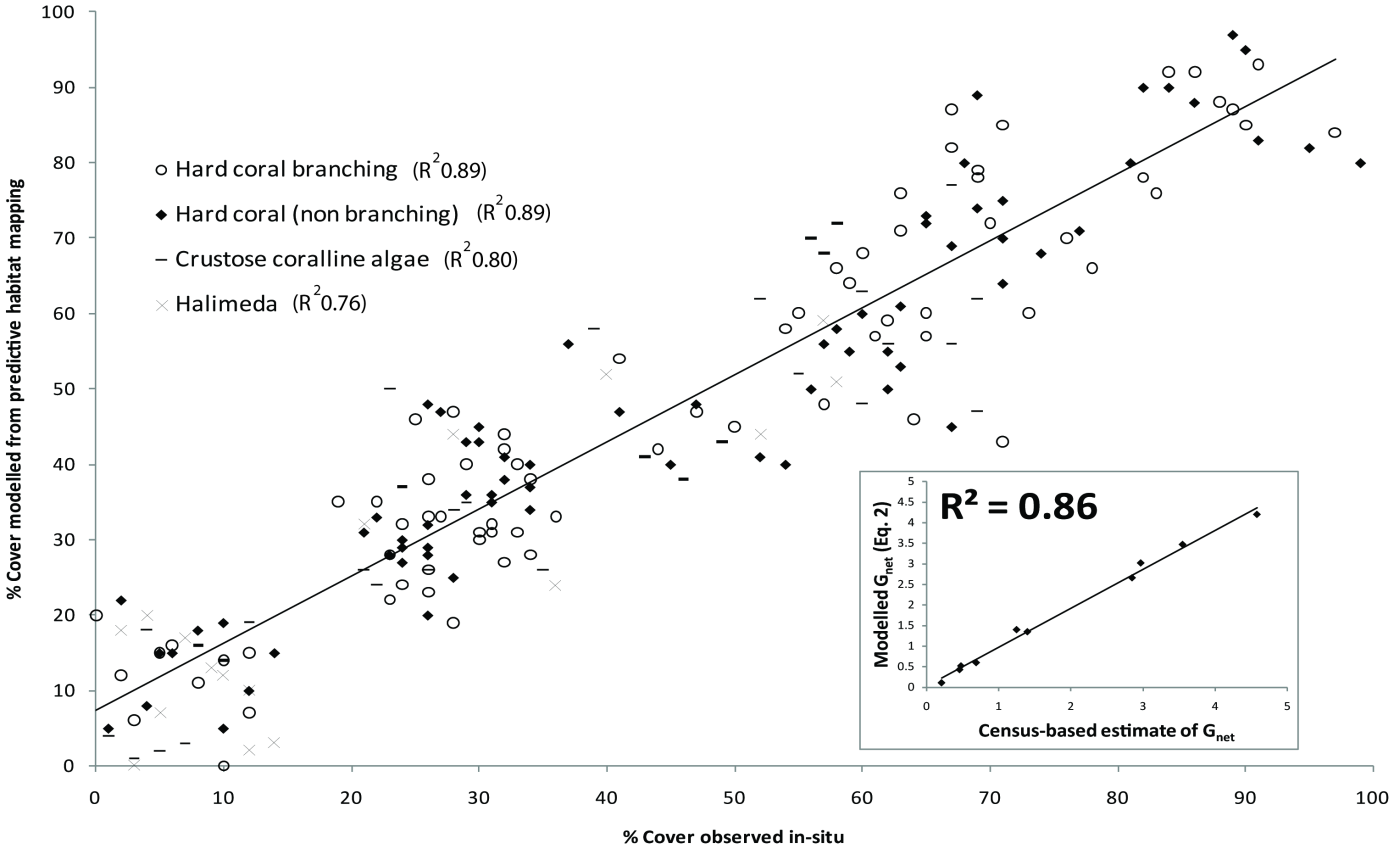
Validation of the maps depicting key benthic carbonate producers for 218 ground reference survey points. Symbology indicates dominant carbonate producer for each survey point. Inset: Validation of net community calcification models using census based estimates from in-situ surveys (Perry et al., 2012).

Benthic carbonate production maps correlated well with independent field validation (mean R^2^ = 0.81), with individual cover correlations as follows: hard coral branching (R^2^ 0.89), hard coral non-branching 0.89, crustose coralline algae (R^2^ 0.8) and *Halimeda* (R^2^ 0.76) (Figure 5). *In-situ* estimates of carbonate production ranged from 0.21 to 4.58 kgCaCO_3_m-^2^yr^−1^ (Table 2). These values correlated well with those predicted from inorganic aragonite precipitation using the rate laws (R^2^ = 0.86, inset Figure 5).

**Table 2:** Census-based estimates of reef community carbonate production for validation transects around the reef platform at Lady Elliot Island (for transect locations, see Figure 1A).

Site	Rugosity, k^′^_r_	*G_net_* (kgCaCO_3_/m^2^/yr)	Live coral cover % (±St.Dev)	Crustose coralline algae cover % (±St.Dev)	Halimeda cover % (±St.Dev)	Dominant substrate type
1 L-shaped reef (site #1)	1.18	1.25	30.7±14.49	11.2±5.64	6.75±1.84	Hard coral (encrusting)
1 L-shaped reef (site #2)	1.22	1.40	51.2±1.11	12.6±2.14	7.28±1.22	Hard coral (branching)
2 Spider (site #1)	1.37	0.69	14.6±6.36	8.3±1.36	2.21±1.27	Dead coral
2 Spider (site #2)	1.54	0.48	23.6±1.77	21.5±4.21	4.31±2.01	Hard coral (branching)
3 Second Reef (site #1)	1.47	4.58	44.4±4.95	9.46±2.06	5.21±1.83	Hard coral (branching)
3 Second Reef (site #2)	2.08	3.55	32.1±8.35	12.14±1.92	7.9±2.62	Sand
4 Hiros Cave (site #1)	1.73	2.97	22.6±2.21	31.2±4.12	0±0	Crustose coralline algae
4 Hiros Cave (site #2)	1.49	2.85	18.2±1.48	38.6±3.08	8.4±1.2	Crustose coralline algae
5 Blow Hole (site #1)	1.49	0.46	12.7±2.09	18.2±9.12	13.94±2.7	Turf algae
5 Blow Hole (site #2)	2.16	0.21	12.6±0.03	15.9±6.21	57.8±11.2	*Halimeda*

**Figure 6:**
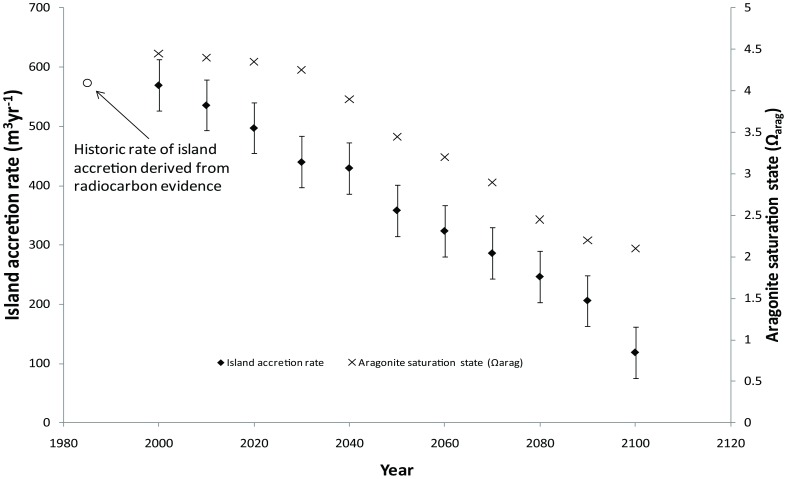
Simulated reductions in reef flat aragonite saturation state and island accretion rates for Lady Elliot Island, 2000–2100.

The reef platform was estimated to generate a total of 569 m^3^ yr^−1^ of sediment. Over the simulation period from 2000 until 2100, this decreased by 79% to 118 m^3^ yr^−1^ because of the reduced aragonite saturation states associated with the RCP8.5 emissions scenario. While the aragonite saturation states decreased in an exponential fashion, simulation results indicated a linear decrease in reef scale carbonate production (Figure 6). A forward extrapolation suggests that the island would enter an erosive state in 2150, assuming other conditions (e.g. reef benthic cover) remained constant.

**Figure 7:**
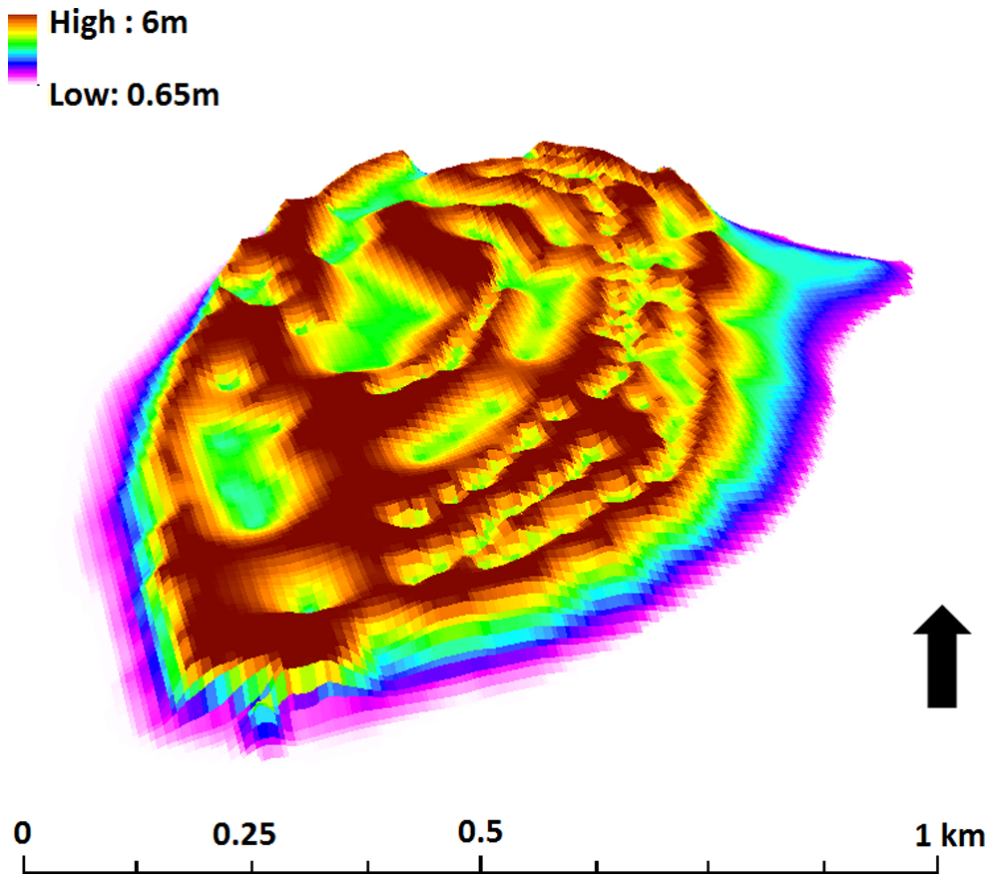
A digital elevation model of Lady Elliot Island generated from a dGPS survey. The north-south aligned shingle ridges are evident.

Figure 7 illustrates the digital elevation model of the island generated with the dGPS survey points, from which the total volume of Lady Elliot Island was calculated to be 1.83 km^3^. Although it is likely that the island was deposited in a pulsed fashion, it is possible to infer from radiocarbon evidence of the time of initial deposition 3200 years ago [29], that the average rate of island volume increase has been approximately 573 m^3^ yr^−1^ since growth was initiated on the reef platform.

## Discussion

The relationship between reef community calcification and aragonite saturation state is not straightforward due to complexities such as the species-specific internal up-regulation of pH in scleractinian corals [56], confounding effects of nutrient input [24] and the buffering capacity of other reef dwellers, such as sea cucumbers [57]. Nevertheless, evidence of the general relationship between coral reef net community calcification and ocean acidification exists for several sites globally, e.g. the Red Sea, Ningaloo, Hawaii, Bermuda, the Gilf of Eilat, Palau, Okinawa Moorea etc. (for a review, see [18]). In the present study, the scaled up carbonate production rates derived using the hydrochemical measurements and satellite remote sensing imagery to generate community coverage and rugosity parameters for the rate law equations resulted in remarkably similar contemporary rates of island accretion to those estimated historically (from 3200 yrs B.P. to present) using radiocarbon evidence. These were estimated to be 569 m^3^ yr^−1^ in the former, compared to 573 m^3^ yr^−1^ in the latter. While similar volumes of island accretion have been modelled by the two different approaches, this does not necessarily translate into consistent rates of island growth over the time period modelled. Evidence of a greater historical capacity to produce carbonate around the reef slope was observed in the form of many dead coral bommies. Physical laws also indicate that the volume of shingle in the island is roughly proportional to the square of the radius of the island, which suggests that to maintain a constant rate of growth, the rate of accretion must have increased with island growth. Further evidence of variable rates of island accretion is provided by shingle ridges, which indicate episodic island evolution whereby accretion occurs in conjunction with high energy events, such as storms and cyclones [29].

Although Shaw et al. [52] demonstrate that diurnal variability in water overlying the reef flat results in a highly non-linear, threefold amplification of pCO2 by the end of the century, these exponential amplifications do not translate into reductions of sediment availability for island accretion, which shows a linear decrease. This is perhaps because of the comparatively large contribution of carbonate produced on the reef slope [58], which dampens the signal of reductions in calcification occurring on the ponded reef flat. Nevertheless, by 2100 simulations suggest that the sediment available for island accretion will reduce to 118 m^3^ yr^−1^, a marked reduction from current levels of 570 m^3^ yr^−1^. Given the consistent downward trajectory, Lady Elliot Island is likely to enter an erosive regime by the year 2150, assuming constancy in other environmental conditions.

The simulations presented have bleak implications for reef island development, particularly because they assume constancy in the nature of the benthic community inhabiting the reef platform around Lady Elliot island, which represents a best case scenario for carbonate production levels. Substantial spatial and temporal variation occurs within reef biota across different reef sub-environments and while the use of spatially extensive remote sensing datasets presented here is effective for capturing the former, it does not capture the latter. This could be achieved by repeating the mapping exercise at appropriate time intervals (e.g. decadal). Furthermore, reef island development is a function of interrelationships between reef basement, sediment supply and hydrodynamic process regimes that drive geomorphic fluctuations across centennial timescales [59]. This study could be profitably improved by accounting more explicitly for processes responsible for sediment generation from carbonate framework, variation in sediment grain size and incident wave energy. It is the combination of these factors that determines sediment transport from the ‘biological factory’ site of production on the reef slope and platform periphery to the site of deposition [22]. For example, the reef energy window index could be incorporated to generate a physically meaningful descriptor of the efficacy of geomorphic processes on reefs by accounting for reef flat width and water depth [60]. At Lady Elliot island, this has been shown to accurately capture gross differences in shoreline character (gradient) caused by the wave energy reaching shorelines at different aspects, with a low index on the windward coast (wide reef flat, small sediment size) and a high index on the leeward coast (narrow reef flat, coarse sediments) [60]. Given additional information on sediment transport dynamics, it may be possible to further extend simulations to generate future estimations of island boundary locations.

Many studies of the impact of ocean acidification on coral reefs employ carbonate chemical parameters from oceanic as opposed to coastal settings (e.g. [61]). However, the presence of shallow water biogenic communities such as coral reefs leads to modifications in seawater carbonate chemistry which should be accounted for when considering the impacts of ocean acidification on community carbonate production [54], [62]. By employing independent values for carbonate chemistry, temperature and salinity conditions over a 7 hour tidal cycle, it was possible to account for fluctuations in seawater chemical properties due to reef flat metabolic processes (photosynthesis, respiration, calcification and dissolution) and air-sea CO_2_ fluxes, to generate a realistic chemical scenario for the projections. This is particularly important at Lady Elliot Island because the water ponds over the reef flat at low tide, leaving it effectively isolated from offshore conditions for approximately 2–3 hours each tidal cycle, when extreme carbonate chemistry conditions occur [29]. These fluctuations influence the chemistry at many sites on the Great Barrier Reef, particularly those where reduced hydrodynamic flushing combined with anthropogenic input from coastal development (for example, the addition of nitrates and phosphates) might also influence carbonate chemistry [13], [24], [25].

## Conclusions

The present study uses rate laws to generate estimates of total reef system carbonate production by combining spatially extensive remotely sensed information on community composition and rugosity with local hydrochemical measures of seawater carbonate chemistry. Estimates of carbonate production are constrained by realistic values and model outputs are coupled with reconstructions of historical island evolution from radiocarbon dates. This approach draws on the increasing suitability of remote sensing images to accurately and consistently upscale estimates of carbonate production from observable features, such as live coral [39]. The inclusion of topographic complexity through the high resolution digital elevation model enabled a more realistic quantification of community carbonate production than has hitherto been the case with two dimensional digital representations of benthic communities [63].

A linear decrease in carbonate production has been simulated at the scale of the complete reef system, with a 79% reduction on current levels by 2100. This is consistent with documented declines in carbonate production elsewhere. Perhaps the most valuable and novel aspect of this study is that it has demonstrated how several disparate techniques can be combined to provide a deterministic means of modelling changes in reef carbonate production and consequent implications for island evolution through time.
